# CRISPR/Cas9-Driven *Fndc5* Knockout Reveals Augmented Mitochondrial Structural and Dynamic Alterations in Diabetic Nephropathy

**DOI:** 10.7150/ijms.131007

**Published:** 2026-07-30

**Authors:** Chien-Wei Huang, Hsin-Hung Chen, Tzu-Ming Jao, Chia-Jung Li, Junne-Ming Sung, Yau-Sheng Tsai, Jin-Shuen Chen

**Affiliations:** 1 Institute of Clinical Medicine, College of Medicine, National Cheng Kung University, Tainan, Taiwan.; 2 Division of Nephrology, Department of Internal Medicine, Kaohsiung Veterans General Hospital, Kaohsiung, Taiwan.; 3 School of Medicine, National Yang Ming Chiao Tung University, Taipei, Taiwan.; 4 Department of Medical Education and Research, Kaohsiung Veterans General Hospital, Kaohsiung, Taiwan.; 5 Global Innovation Joint Degree Program, International Joint Degree Master's Program in Agro-Biomedical Science in Food and Health, College of Medicine, National Taiwan University, Taipei, Taiwan.; 6 Department of Obstetrics and Gynaecology, Kaohsiung Veterans General Hospital, Kaohsiung, Taiwan.; 7 Institute of Biopharmaceutical Sciences, National Sun Yat-sen University, Kaohsiung, Taiwan.; 8 Division of Nephrology, Department of Internal Medicine, National Cheng Kung University Hospital, College of Medicine, National Cheng Kung University, Tainan, Taiwan.; 9 Institute of Precision Medicine, National Sun Yat-Sen University, Kaohsiung, Taiwan.

**Keywords:** *Fndc5*, irisin, mitochondrial morphology, mitochondrial dynamics, oxidative stress, diabetic nephropathy

## Abstract

FNDC5 has been implicated in glucose homeostasis and is associated with mitochondrial function. Its role in diabetes and diabetic nephropathy (DN) remains unclear. This study hypothesizes that FNDC5 deficiency predisposes the kidney to accelerated mitochondrial dysfunction in diabetes and DN. Systemic *Fndc5* knockout (KO) C57BL/6 mice were generated using CRISPR/Cas9. DN were induced in six-week-old *Fndc5* wild-type (WT) and KO mice using high-fat diet combined with streptozotocin injection. Weekly blood and urine analyses assessed glucose, cholesterol, triglycerides, blood urea nitrogen, creatinine, and proteinuria. At 15 weeks, kidneys and metabolic tissues including pancreas, muscle and adipose were collected for histological and molecular analyses. Results showed that while both *Fndc5* WT and KO mice were successfully induced with hyperglycemia, the *Fndc5* KO DN group exhibited a slightly lower cumulative glycemic burden compared with the WT DN group. Despite this milder metabolic stress, proteinuria remained comparable between the two groups. Furthermore, histological analysis revealed that *Fndc5* KO DN mice displayed more severe mesangial expansion, glomerular basement membrane thickening, and podocyte effacement compared with WT DN mice. The elevated lipid peroxidation, reduced PGC-1α expression, and increased DNA fragmentation were also evident in *Fndc5* KO DN. More swollen mitochondria with a significantly higher percentage of disrupted cristae were observed in *Fndc5* KO DN mice compared with WT DN mice. This was accompanied by the upregulation of mitochondrial fission-related genes (*Dnm1l* and *Fis1*), downregulation of the fusion-related gene (*Mfn1*), and reduced expression of ATP synthase subunits (ATP5A1 and ATP5B). Systemic analysis of other metabolic tissues, including the pancreas and muscle and adipose tissues, revealed increased lipid peroxidation and decreased PGC-1α expression. These findings underscore FNDC5's role in maintaining mitochondrial integrity and cellular health under diabetic conditions, positioning FNDC5 as a potential therapeutic target.

## Introduction

Diabetes, characterized by insulin resistance and hyperglycemia, represents a major global health challenge with substantial morbidity and mortality 1. Among its complications, diabetic nephropathy (DN) is a leading cause of end-stage kidney disease (ESKD) 2. Understanding the complex molecular mechanisms underlying DN is crucial for effective therapeutic strategies. Mitochondrial dysfunction plays a central role in the pathophysiology of diabetes and DN, contributing to both insulin resistance and deficiency 3, 4. In diabetes, impaired mitochondrial coupling and excessive reactive oxygen species (ROS) production lead to oxidative damage, further exacerbating insulin resistance 5, 6. This dysfunction is particularly evident in insulin-sensitive tissues, such as skeletal muscle, white adipose tissue, and liver, where diminished mitochondrial function further predisposes individuals to ectopic lipid accumulation 7. Furthermore, mitochondrial dysfunction contributes to DN by increasing oxidative stress via excessive production of ROS and impaired oxidative phosphorylation 4. Elevated ROS levels initiate cascades of inflammation, cellular damage, and apoptosis, ultimately resulting in kidney fibrosis and progressive kidney function decline. Collectively, these findings underscore the pivotal role of mitochondrial function in the pathophysiology of diabetes and DN, emphasizing the potential of targeting mitochondrial pathways as therapeutic interventions 8.

Irisin, a myokine derived from the extracellular domain of fibronectin type III domain-containing protein 5 (FNDC5), has garnered significant attention for its role in glucose homeostasis and metabolic regulation 9-12. Studies have shown that irisin promotes the browning of white adipose tissue, enhances thermogenesis, and improves systemic lipid metabolism 13. Irisin also modulates obesity-induced inflammation, as demonstrated in high-fat diet-induced obese mice. *Fndc5* gene deletion exacerbates obesity, insulin resistance, and inflammation, while its overexpression attenuates these conditions through AMPK-mediated macrophage polarization 14. For diabetes, clinical studies have reported that diabetic patients have lower serum irisin levels compared with healthy controls 15, 16. Additionally, patients with diabetes and DN exhibit even lower serum irisin levels compared with those without DN 17. Nevertheless, the precise role of irisin in DN remains elusive. Furthermore, mitochondrial biogenesis has been proposed as a potential signalling pathways which irisin exerts its effects in myocytes and adipocytes 11. However, the influence of irisin on mitochondrial function in diabetes and DN remains unclear.

This study aims to elucidate the role of FNDC5 in diabetes and DN by investigating the systemic and molecular effects of FNDC5 deficiency in a mouse model. We employed CRISPR/Cas9 technology to generate global *Fndc5* knockout (KO) mice on a C57BL/6 background and established DN by combining a high-fat diet with low-dose streptozotocin (STZ) injection, a well-established approach for modeling diabetes related kidney injury. We analyzed metabolic responses, pathophysiological changes, cellular and mitochondrial functions in various tissues, with a particular focus on the kidneys. Our findings reveal that FNDC5 deficiency is associated with aggravated mitochondrial injury characterized by structural disintegration and dynamic alterations in kidney tissues, leading to heightened oxidative stress, DNA damage, and aggravated glomerular injury. Additionally, significant systemic effects were observed in metabolic tissues, including the pancreas, muscle, brown adipose tissue, and white adipose tissues, underscoring the critical role of FNDC5 in maintaining metabolic homeostasis under diabetic conditions. These insights highlight the potential of FNDC5 as a therapeutic target for mitigating diabetes and DN complications.

## Methods

### Animal Housing and Care

*Fndc5* wild-type (WT) and KO C57BL/6 mice (total: 36; age: six weeks; weight: 16-25 g; 15 females and 21 males) were used in the study. The cohort consisted of 7 WT and 6 *Fndc5* KO mice assigned to the control groups, and 10 WT and 13 *Fndc5* KO mice subjected to the diabetic induction protocol. Detailed records regarding the diabetic induction success rate and the subsequent longitudinal survival numbers are provided in **Fig. S1**. Systemic C57BL/6JNarl-Fndc5^em1Jsc^ mice were generated using CRISPR/Cas9 gene-editing technology (Supplementary Method 1). The project involved designing and producing *Fndc5* exon2 and exon3 KO. The mice were purchased from The Jackson Laboratory and backcrossed to BALB/cByJNarl strain (National Laboratory Animal Center, Taipei, Taiwan). Tail tip tissue samples (approximately 1 mm) were collected from 4-week-old mice under isoflurane anesthesia (4-5% for induction and 1-3% for maintenance) for PCR-based genotyping to confirm the presence of the targeted genes. Animal experiments were conducted in strict adherence to the standards and procedures approved by the Ethics Committee for Animal Care and Use at Kaohsiung Veterans General Hospital, Kaohsiung, Taiwan (Approval No: IACUC-2022-2023-A038). All procedures conformed to the institutional guidelines for animal experimentation. Mice were housed in the specific pathogen-free facility of the Laboratory Animal Center at Kaohsiung Veterans General Hospital. They were maintained in individually ventilated cages with positive pressure, at a controlled ambient temperature of 20-25 °C and humidity levels of 50-60%. A 12-hour light/dark cycle was strictly enforced. The mice were provided with unlimited access to standard laboratory chow and water. To ensure animal welfare throughout the study, humane endpoints were established based on specific clinical signs, including significant weight loss, loss of appetite, severe infection, clinical signs of severe organ dysfunction unresponsive to treatment, and moribund or near-death condition. Euthanasia was performed by 30% vol/min CO2 absorption. Death was confirmed by cardiac arrest, respiratory arrest, absence of blink response, and lack of pain reflex, with an observation period of 3 minutes. If death was uncertain, cervical dislocation was performed to ensure euthanasia.

### Induction of DN Mouse Models

For the induction of DN, six weeks-old C57BL/6 mice with *Fndc5* WT and KO were randomly divided into two groups: the control group and the DN group. The control group was fed a normal-fat diet for 10 weeks (Altromin 1310, Supplementary Method 2), while the DN group was fed a high-fat diet for 10 weeks (D12492, Research Diets, Supplementary Method 3). At seven weeks of age, the mice in the DN group were fasted for 12 hours before being induced to develop DN. They were intraperitoneally injected with 100 mg/kg body weight STZ (S0130, Sigma-Aldrich) in 0.1 M citrate buffer (pH 4.5). The control group, serving as the normal control group, received an intraperitoneal injection of 0.1 M citrate buffer (pH 4.5) at seven weeks of age. Fasting blood glucose levels were monitored the following week using blood samples (100 μL) collected from the retro-orbital plexus under isoflurane anesthesia (4-5% for induction, 1-3% for maintenance), and mice with blood glucose concentrations exceeding 200 mg/dL were classified as diabetic. The retro-orbital sinus was chosen as the site of blood collection due to its ability to yield sufficient blood volume with minimal stress and no significant long-term adverse effects 18. To prevent complications, the site was alternated weekly between the eyes. To assess the development and progression of DN, the urine protein to creatinine ratio (uPCR) was measured weekly to confirm the onset and quantify the severity of proteinuria.

### Biochemical Assays of Blood and Urine

Blood and urine samples were collected, microcentrifuged, and stored at -80°C until analysis. Blood samples (100 μL) were collected weekly from the retro-orbital plexus under isoflurane anesthesia. Fasting blood glucose levels were monitored using the Accu-Chek Instant system (Roche). The maximum weight loss induced by fasting was 5.6%. After centrifugation, serum was separated and stored. Serum concentrations of blood urea nitrogen (BUN), creatinine, cholesterol, and triglycerides were measured using the Ortho-Clinical Diagnostics VITROS 350 System. Urine samples (0.5-1.5 mL) were collected weekly using metabolic cages (SN-783, Hinano). Urinary protein concentration was quantified using a bicinchoninic acid protein assay kit (Pierce), and proteinuria was calculated as the ratio of urinary protein (mg/mL) to urinary creatinine (mg/dL). Urine creatinine levels were measured using the Ortho-Clinical Diagnostics VITROS 350 System. All assays were performed in duplicate according to the manufacturer's instructions.

### Sample Collection and Tissue Preparation

Body weight was recorded weekly to monitor changes in the mice. At the end of the experiment, when the mice were 15 weeks old, samples of blood, pancreas, kidneys, muscle, and adipose tissues were collected following mice euthanasia. Blood samples were centrifuged and stored at -80 °C until analysis. Tissue samples were quickly frozen and stored at -80 °C. Additionally, some tissue samples were fixed in 10% formalin, dehydrated, and embedded in paraffin blocks for preservation.

### RNA Extraction and Real-Time Reverse-Transcription Polymerase Chain Reaction

Total RNA was extracted from mouse kidneys using RNAzol® RT. A small piece of tissue was placed into a 2 mL microcentrifuge tube containing 1000 μL of RNAzol® RT and homogenized using tissue grinding beads. After adding 400 μL of sterile water, the mixture was incubated at room temperature for 3 minutes and then centrifuged at 13,000 g for 15 minutes. The supernatant (1000 μL) was transferred to a new tube and mixed with an equal volume of isopropanol by gentle inversion, followed by a 10-minute incubation at room temperature. The mixture was centrifuged at 13,000 g for 10 minutes at 4 °C. The supernatant was discarded, and the RNA pellet was washed with 500 μL of 75% ethanol, centrifuged at 4,000 g for 5 minutes at 4 °C. This wash step was repeated twice. The remaining ethanol was removed, and the RNA pellet was air-dried. The RNA was resuspended in sterile water, and its concentration was measured using a spectrophotometer. Subsequently, 500 ng of total RNA was reverse-transcribed into complementary DNA (cDNA) using the PrimeScript RT Master Mix (Takara) following the manufacturer's instructions. The resulting cDNA was used as a template for real-time polymerase chain reaction (PCR). All samples were stored at -20°C until further analysis. Messenger RNA levels of genes in this study, including *Fndc5*, *Dnm1l*, *Mfn1*, and *Fis1*, were determined by real-time reverse-transcription PCR using the iQ™ SYBR® Green Supermix (Bio-Rad Laboratories), following the manufacturer's instructions. Quantitative analysis of glyceraldehyde-3-phosphate dehydrogenase (GAPDH) expression was performed for each sample to serve as an internal control, allowing for the calculation of relative gene expression levels.

### Western Blot Analysis

Cell lysates were prepared using RIPA buffer (pH 7.6) with protease and phosphatase inhibitors, followed by centrifugation at 14,000 g for 30 minutes. Protein concentration was determined, and 20 μg of protein was separated on a 12% SDS-PAGE and transferred onto PVDF membranes. Membranes were blocked with 5% milk in TBST for 1 hour, then incubated with primary antibodies (nephrin, 1:1000 [Proteintech, 22912-1-AP]; podocin, 1: 1000 [ABclonal, A17337]; ATP5A1, 1:5,000 [ABclonal, A11217]; ATP5B, 1:10,000 [ABclonal, A11214]; β-actin, 1:10,000 [GeneTex, GTX109639]) at 4 °C overnight. After incubation with HRP-conjugated secondary antibodies, signals were detected using chemiluminescent substrate and visualized with an imaging system. Quantification was done using Image Lab software.

### Histology and Immunohistochemistry (IHC) Staining

Mouse kidneys were preserved in 10% neutral-buffered formalin, dehydrated, and embedded in paraffin. Periodic acid-Schiff (PAS) staining was used to evaluate the condition of the glomerular basement membrane, mesangial matrix and kidney tubule. Tissue sections (2 μm thick) were cut from paraffin-embedded blocks and baked at 60 °C for 24 hours. Sections were then deparaffinized with xylene and rehydrated through a graded series of ethanol (100%, 95%, 75%) to 1X PBS. The sections were stained using the ScyTek Periodic Acid Schiff (PAS) Stain Kit. First, sections were incubated with PAS solution for 10 minutes and rinsed four times with sterile water. Next, Schiff solution was applied for 30 minutes, followed by a rinse with running warm water and one rinse with sterile water. Hematoxylin was then applied for 4 minutes to counterstain the nuclei, followed by a 2-minute rinse with running water. Bluing reagent was applied for 10 seconds, and the sections were washed once with sterile water. Finally, sections were dehydrated through a graded series of ethanol (75%, 95%, 100%), air-dried, and mounted with coverslips. For IHC staining, tissue sections (2 μm thick) were cut from paraffin-embedded blocks and baked at 60 °C for 24 hours. Sections were then deparaffinized with xylene and rehydrated through a graded series of ethanol (100%, 95%, 75%) to 1X PBS. Antigen retrieval was performed by placing the sections in citrate buffer (pH 6.0) and heating in a pressure cooker for 20 minutes, then cooling to room temperature. Subsequent staining was carried out using the Leica Novolink Polymer Detection Systems kit according to the manufacturer's instructions. The target proteins (FNDC5 [Proteintech, 23995-1-AP], 4-hydroxynonenal [4-HNE, Bioss, bs-6313R], and Peroxisome proliferator-activated receptor gamma coactivator 1-alpha [PGC-1α, Abclonal, A12348]) were stained using this protocol. Finally, the sections were dehydrated through a graded series of ethanol (75%, 95%, 100%), air-dried, and mounted with coverslips. Quantitative analysis of IHC staining was performed using ImageJ software. For each kidney section, 5-10 nonoverlapping fields were randomly captured at 400× magnification. The H-score was averaged across these fields to provide a single representative value for each animal.

### Terminal Deoxynucleotidyl Transferase dUTP Nick End Labeling (TUNEL) Assay

TUNEL positivity in kidneys from WT and *Fndc5* KO mice was determined using the In Situ Cell Death Detection Kit, POD (Roche). Kidney tissue sections (2 μm thick) were prepared from paraffin-embedded blocks and baked at 60 °C for 24 hours. Sections were then deparaffinized with xylene and rehydrated through a graded series of ethanol (100%, 95%, 75%). Following rehydration, sections were placed in citrate buffer (pH 6.0) and microwaved at 350 W for 5 minutes. After washing with 1X PBS, TUNEL reaction mixture was added, and sections were incubated at 37 °C for 1 hour in the dark. Post-incubation, sections were washed and counterstained with DAPI for 5 minutes. Finally, sections were washed with 1X PBS and mounted with coverslips. For quantification, a minimum of 5 random fields per kidney were analyzed under 400× magnification.

### Electron Microscopy

The kidneys were preserved in 2.5% glutaraldehyde and embedded with the Low Viscosity Embedding Media Spurr kit (Sigma-Aldrich). For ultrastructural examination, kidney tissue sections of 50-70 nm thickness were examined using a transmission electron microscopy (JEM 1400 PLUS; JEOL). To quantify mitochondrial cristae, six non-overlapping fields of view were randomly captured at high magnification for each sample to ensure unbiased sampling. Within these fields, at least 50 mitochondria per mouse were analyzed. Mitochondrial cristae, defined as invaginations of the inner mitochondrial membrane extending into the matrix, were manually counted. The resulting cristae counts were subjected to statistical analysis to compare the mean number of cristae per mitochondrion among experimental groups.

### Statistical Analysis

Data from male and female mice were pooled for analysis and presented as the mean ± standard error of the mean (SEM). The number of independent biological replicates (n = 3 to 6 mice per group) for each experiment is specified in the corresponding figure legends. All histological, immunohistochemical, and ultrastructural assessments were conducted by investigators blinded to the experimental groups to ensure objectivity. Before statistical comparison, the Shapiro-Wilk test was used to assess data normality. For normally distributed data, comparisons between two groups were performed using a two-tailed Student's t-test, while the Mann-Whitney U test was applied for non-normally distributed data. For comparisons across multiple groups, a two-way analysis of variance (ANOVA) was conducted, followed by Tukey's post-hoc test to correct for multiple comparisons. For all histological and IHC quantifications, the unit of analysis was the individual animal, with the value for each animal representing the mean derived from 5-10 randomly selected fields. All tests were 2-sided with *p* < 0.05. Analyses were performed using GraphPad Prism 9 (GraphPad Software, San Diego, CA, USA). A *p* value < 0.05 was considered statistically significant, with significance levels indicated as *p < 0.05, **p < 0.01, and ***p < 0.001.

## Results

### Mice with and Without Fndc5 KO Exhibit Distinct DN Features After High Fat Diet and STZ Injection

**Figure 1:**
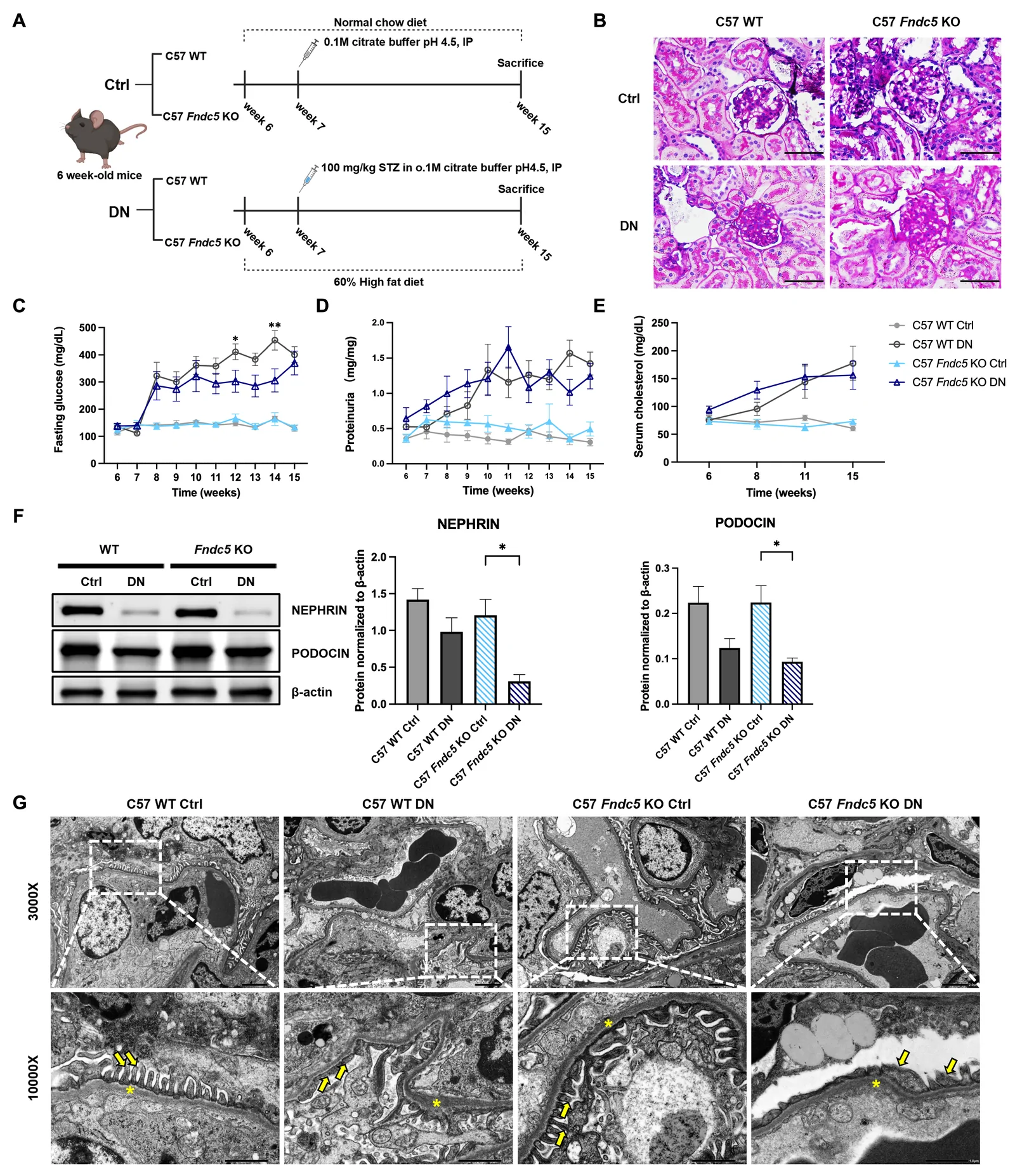
** Impact of FNDC5 deficiency on kidney and metabolic functions in DN mice.** (A) Experimental design for DN induction in C57 WT and *Fndc5* KO mice with control comparisons. (B) Glomerular basement membrane thickening and mesangial expansion were evaluated by periodic acid-Schiff staining. Scale bars: 100 µm. Time course of fasting glucose levels (C), proteinuria (D), and serum cholesterol (E) in C57 WT and *Fndc5* KO mice, with and without DN induction. (F) Representative Western blot images showing nephrin and podocin protein levels in kidney samples from WT and *Fndc5* KO mice under control and DN conditions, with corresponding quantification normalized to β-actin. (G) Representative transmission electron microscopy images of kidney glomeruli in C57 WT, C57 WT DN, and *Fndc5* KO DN mice, showing ultrastructural alterations in podocyte foot processes and the glomerular basement membrane. Yellow arrows indicate podocyte foot processes, and yellow asterisks indicate the glomerular basement membrane. Scale bars: 2 μm (low magnification) and 1 μm (high magnification). Data are presented as mean ± SEM (n= 4 to 7 for each group). *P < .05; **P < .01; ***P < .001.

We first established a DN model in C57 WT and *Fndc5* KO mice using a high-fat diet combined with STZ injection, as illustrated in **Fig 1A**. The success rates of diabetes induction, defined by achieving a blood glucose level exceeding 200 mg/dL one week post-STZ injection, were 90% in C57 WT and 76.9% in *Fndc5* KO mice. Furthermore, among mice that were successfully induced with diabetes, the survival rates at 15 weeks were 66.7% in C57 WT DN and 70% in *Fndc5* KO DN mice (**Fig. S1**). Most deaths occurred within 2 weeks after the mice received a high-fat diet combined with STZ injection, accompanied by markedly elevated blood glucose levels. We used PAS staining to assess the condition of kidney glomeruli, thereby verifying the successful induction of DN (**Fig. 1B**). Compared with C57 WT mice, C57 WT DN mice exhibited significant glomerular basement membrane thickening and notable mesangial expansion, with no marked differences observed in the renal tubules. The *Fndc5* KO DN group showed similar pathological changes to those observed in C57 WT DN mice. Additionally, we employed Masson staining to assess the fibrotic state of the kidneys, and the findings indicated no significant evidence of fibrosis across these groups (**Fig. S2A**). We monitored serum glucose, BUN, creatinine levels and uPCR weekly and compared the differences between groups. The results revealed that serum glucose levels in C57 WT DN mice were significantly elevated one week after STZ injection compared with C57 WT mice, and a similar pattern was observed in *Fndc5* KO DN mice. Notably, longitudinal analysis demonstrated that the *Fndc5* KO DN group maintained a lower glycemic burden compared with the WT DN group throughout the study period, with the difference reaching statistical significance at weeks 12 and 14 (**Fig. 1C**). Starting from the tenth week, the uPCR significantly increased in C57 WT DN mice compared with C57 WT controls, with *Fndc5* KO DN mice showing similar levels to C57 WT DN mice (**Fig. 1D**). The serum BUN and serum creatinine did not show significant differences between the groups (**Fig. S2B** and **Fig. S2C**). Western blot analyses of nephrin and podocin (**Fig. 1F**) showed reduced nephrin protein levels in both DN groups compared with WT controls, with the *Fndc5* KO DN group exhibiting the lowest levels. In contrast, there was no difference in podocin protein levels. The uncropped and unedited full blots were provided in Figure S3. Electron microscopy was employed to compare glomerular morphology among the groups. Compared with C57 WT controls, C57 WT DN mice demonstrated significant glomerular basement membrane thickening and marked podocyte effacement (**Fig. 1G**). Similarly, *Fndc5* KO DN mice exhibited these morphological alterations, consistent with those observed in C57 WT DN mice. These findings confirm successful DN induction in both groups. Additionally, we conducted comparative analyses on metabolic responses in DN mice with and without *Fndc5* KO, specifically examining serum cholesterol, triglycerides, and body weight. From the eleventh week onward, serum cholesterol levels in C57 WT DN mice increased significantly compared with C57 WT mice, while *Fndc5* KO DN mice exhibited serum cholesterol levels similar to C57 WT DN mice (**Fig. 1E**). Nevertheless, other indicators, such as body weight and serum triglycerides, did not show significant differences between the groups (**Fig. S2D** and **Fig. S2E**).

### Lipid Peroxidation, Decreased PGC-1α Expression, and Increased DNA Damage in Kidney Tissue Were Augmented in Fndc5 KO DN Mice

**Figure 2:**
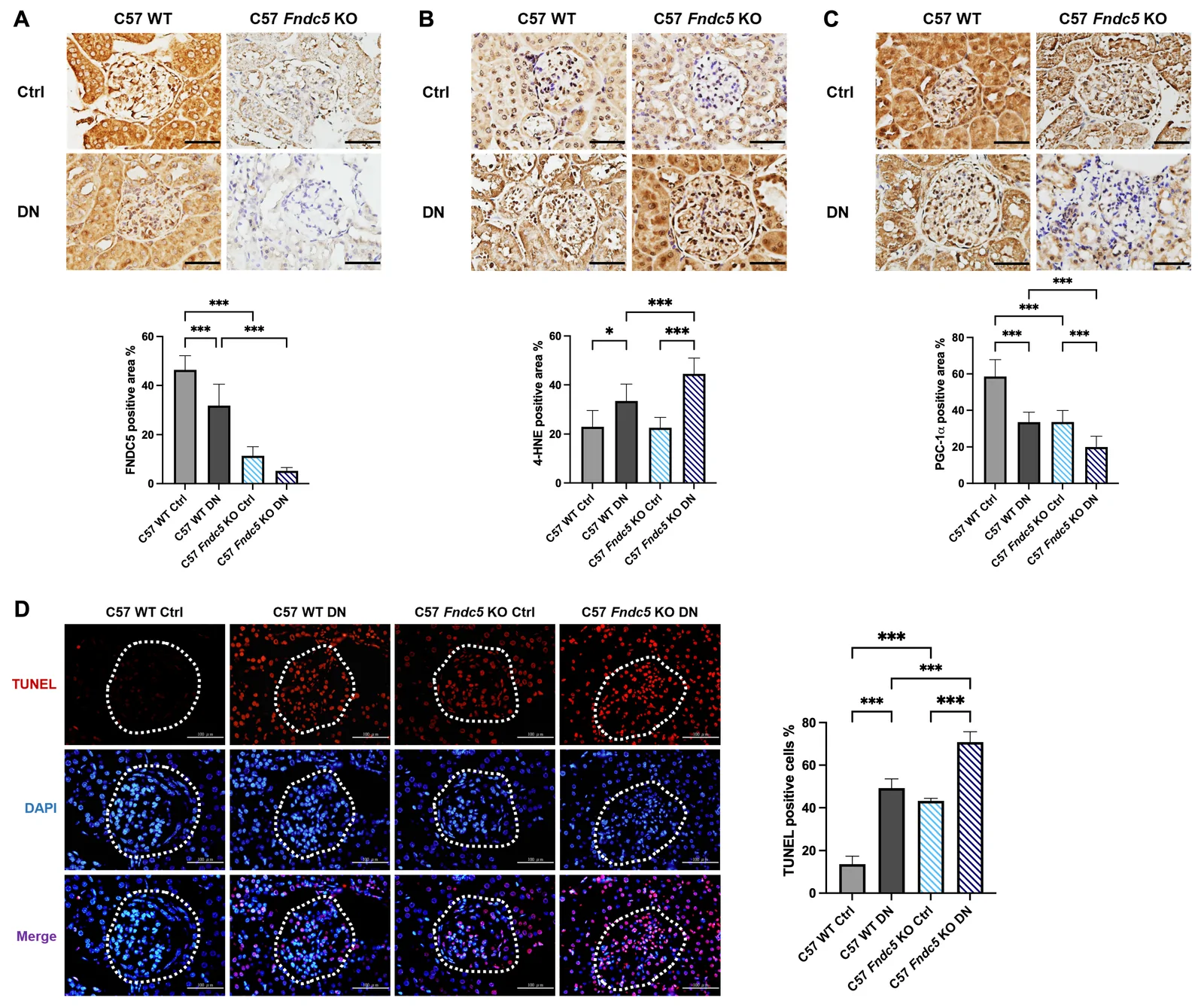
IHC staining and quantitative analysis of FNDC5 (A), 4-HNE (B), and PGC-1α (C) by groups in kidney tissues. Scale bars: 100 µm. The quantitative analysis below each panel represents the percentage of positive staining areas for the respective markers. (D) TUNEL Immunofluorescence staining and quantitative analysis of apoptosis in kidney glomeruli. Scale bars: 100 µm. Data are presented as mean ± SEM (n = 3 to 4 for each group). *P < .05; **P < .01; ***P < .001.

To further investigate the functional impacts of FNDC5 deficiency in DN, we examined markers of lipid peroxidation, PGC-1α expression, and DNA damage in kidney tissues (**Fig. 2**). First, IHC staining confirmed that FNDC5 expression was significantly reduced in both *Fndc5* KO and *Fndc5* KO DN mice compared with C57 WT controls, verifying the ablation of the target gene (**Fig. 2A**). To assess lipid peroxidation indicating the oxidative stress, we analyzed 4-HNE expression in kidney tissue via IHC staining (**Fig. 2B**). The results indicated a significant increase in lipid peroxidation in C57 WT DN mice compared with C57 WT controls, with an even higher level observed in *Fndc5* KO DN mice, suggesting a more significant oxidative burden in the *Fndc5* KO group. We also compared PGC-1α expression levels across the groups (**Fig. 2C**). PGC-1α, a key regulator of mitochondrial biogenesis, oxidative metabolism, and quality control, showed significantly reduced expression in C57 WT DN mice compared with C57 WT controls. Notably, *Fndc5* KO DN mice exhibited a further significant decrease in PGC-1α expression compared with the C57 WT DN group, indicating FNDC5 deficiency was associated with mitochondrial dysregulation in the context of DN. To further assess DNA fragmentation, we conducted a TUNEL assay on kidney tissues (**Fig. 2D**). The assay revealed a marked increase in DNA fragmentation in the kidneys of C57 WT DN mice compared with C57 WT controls. Moreover, the level of DNA fragmentation was significantly higher in *Fndc5* KO DN mice than in C57 WT DN mice. These findings demonstrate that FNDC5 deficiency is linked to more severe oxidative stress, mitochondrial dysregulation, and DNA damage in diabetic kidneys.

### Severe Mitochondrial Injury and Renal Structural Alterations in Fndc5 KO DN Mice

**Figure 3:**
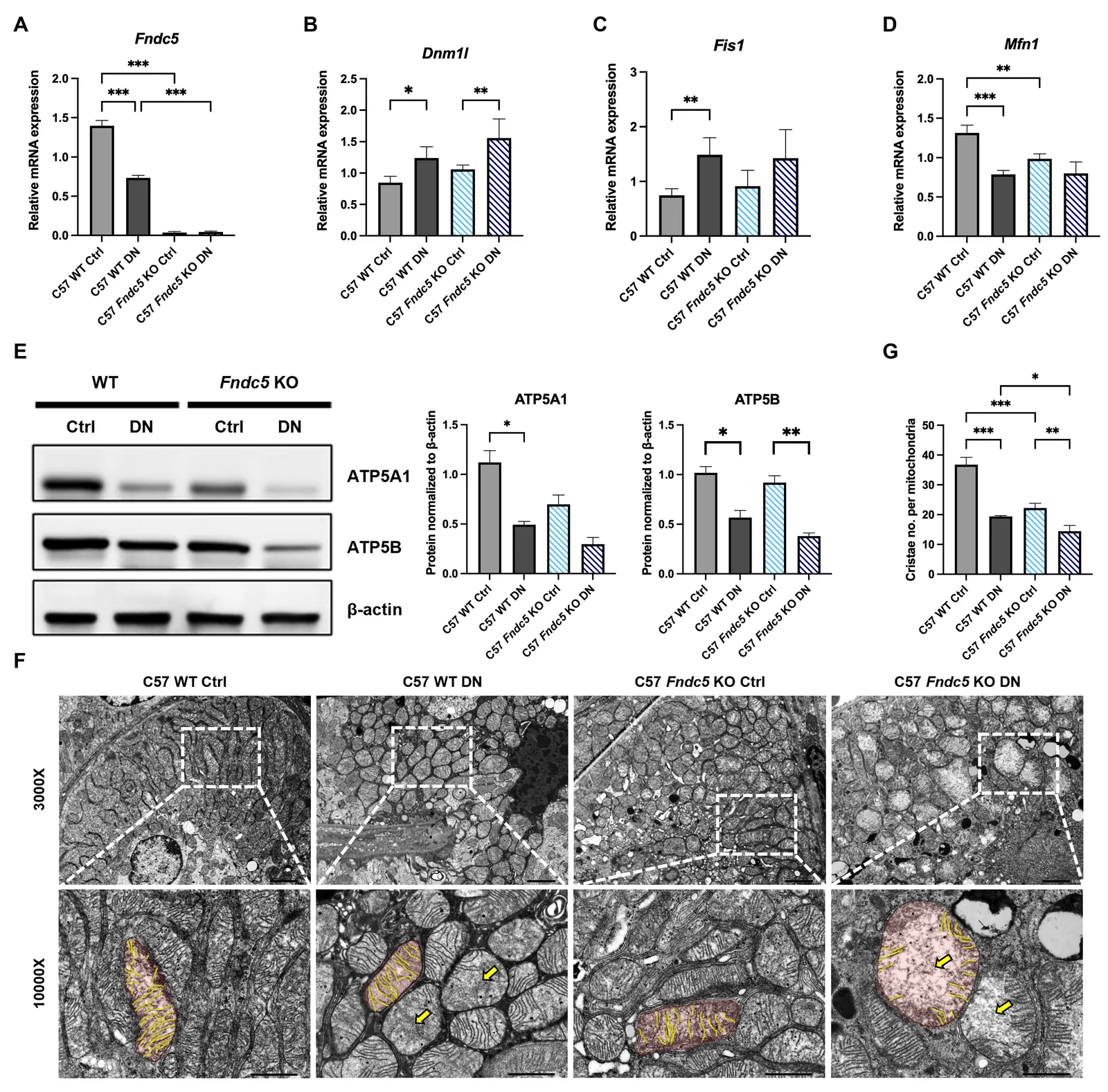
mRNA expression levels of *Fndc5* (A) and mitochondrial function markers, including *Dnm1l* (B), *Fis1* (C), and *Mfn1* (D), in kidney samples from WT and *Fndc5* KO mice, with or without DN induction, normalized to GAPDH. (E) Representative Western blot images showing protein levels of ATP5A1, ATP5B, and β-actin in kidney samples from WT and *Fndc5* KO mice under control and DN conditions, with corresponding quantification normalized to β-actin. (F) Representative transmission electron microscopy images of mitochondrial morphology in proximal tubular cells of WT and *Fndc5* KO mice, with or without DN induction. Red outlines demarcate representative mitochondria, while yellow lines highlight preserved mitochondrial cristae. Yellow arrows specifically indicate mitochondrial swelling characterized by an extensive loss of cristae, most notably in the *Fndc5* KO DN group. Scale bars: 2 μm (low magnification) and 1 μm (high magnification). (G) Quantitative analysis of mitochondrial cristae number per mitochondrion in proximal tubular cells from WT and Fndc5 KO mice, with or without DN induction, based on transmission electron microscopy images. Data are shown as mean ± SEM (n = 4 for each group). *P < .05; **P < .01; ***P < .001.

The mitochondrial function in the kidneys of these DN mice was subsequently evaluated. The mRNA expression of *Fndc5*, *Dnm1l*, *Fis1*, and *Mfn1* in kidney tissue was quantified and normalized to GAPDH (**Fig. 3A-D**). As expected, *Fndc5* expression was significantly reduced in both *Fndc5* KO and *Fndc5* KO DN mice compared with WT mice. *Dnm1l* and *Fis1*, genes associated with mitochondrial fission, showed significantly higher expression in both WT DN and *Fndc5* KO DN groups compared with WT controls, indicating an increase in mitochondrial fission in diabetic conditions. Conversely, *Mfn1*, a gene involved in mitochondrial fusion, was markedly downregulated in both DN groups, suggesting a shift towards fission over fusion in the mitochondrial dynamics of diabetic kidneys. Western blot analyses of ATP synthase subunits ATP5A1 and ATP5B revealed that both DN groups suffered significant protein loss compared to their respective controls (**Fig 3E**). While the *Fndc5* KO DN group displayed the lowest absolute protein levels, the difference between the two DN groups did not reach statistical significance. This suggests that while FNDC5 deficiency is associated with augmented mitochondrial structural disintegration, the depletion of these specific oxidative phosphorylation subunits is a common hallmark of the diabetic state, regardless of genotype, at this stage of disease progression. The full uncropped and unedited versions of the Western blots were presented in **Fig. S4**. Electron microscopy (**Fig. 3F**) provided visual evidence of mitochondrial structural damage in kidney proximal tubules. While WT DN mice displayed disrupted inner mitochondrial membrane cristae, *Fndc5* KO DN mice exhibited more pronounced structural abnormalities, with mitochondria appearing swollen, rounded, and with severe loss of cristae. Quantitative morphometric analysis of the EM images revealed that the number of mitochondrial cristae per mitochondrion was significantly reduced in the *Fndc5* KO DN group compared with the WT DN group (Fig. 3G). These results support that FNDC5 deficiency amplifies mitochondrial structural disintegration and dynamic dysregulation in diabetic kidneys.

### Systemic Impact of Fndc5 KO on Lipid Peroxidation and Mitochondrial Function in Metabolic Tissues of DN mice

**Figure 4:**
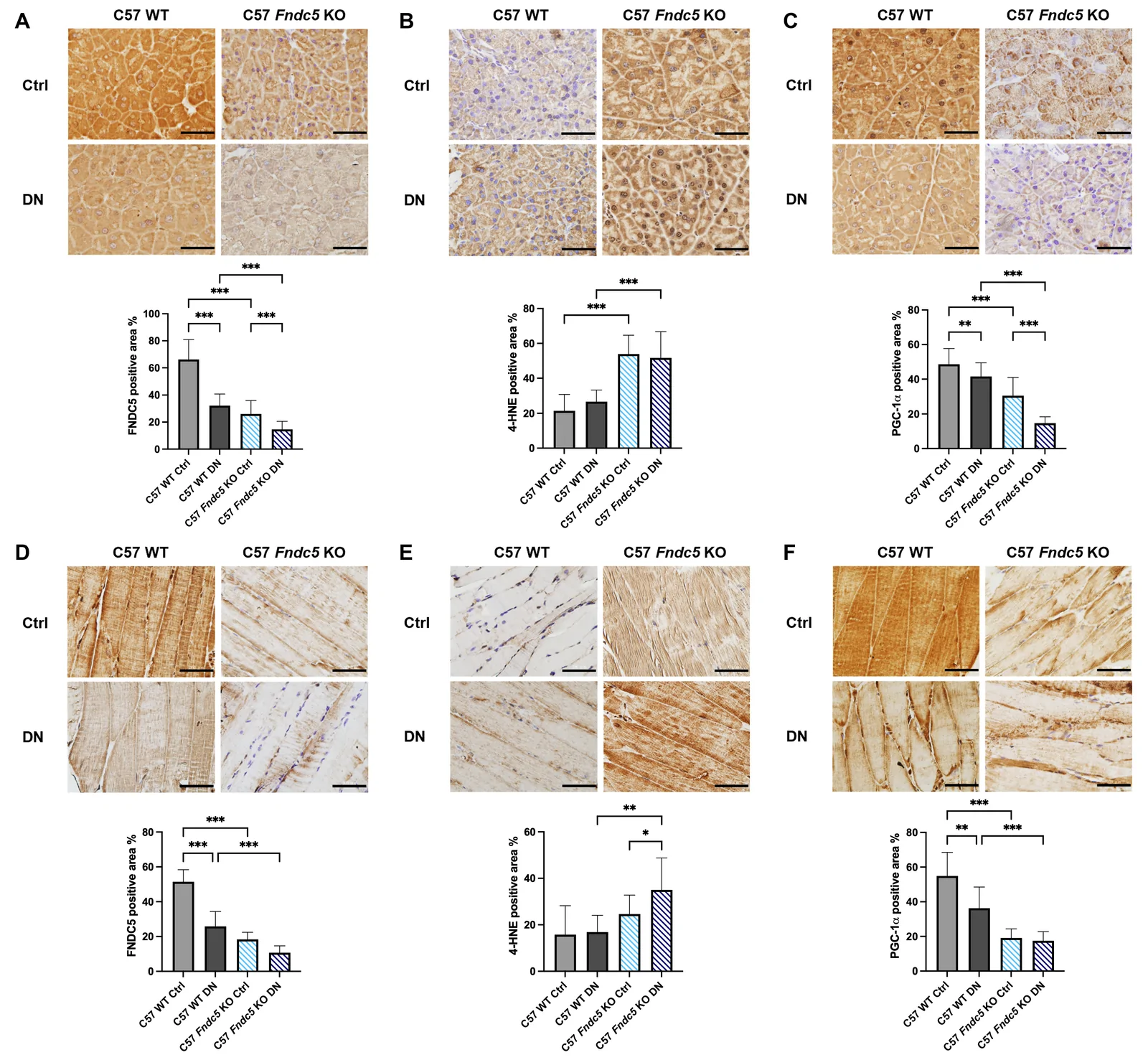
Morphological, IHC, and quantitative analyses of FNDC5 (A, D), 4-HNE (B, E), and PGC1-1α (C, F) in pancreatic and muscle tissues. Panels (A-C) show pancreatic tissue, and panels (D-F) show muscle tissue, with comparisons between WT and *Fndc5* KO groups, both with and without DN induction. Scale bars: 100 µm. Data are shown as mean ± SEM (n = 4 for each group). *P < .05; **P < .01; ***P < .001.

**Figure 5:**
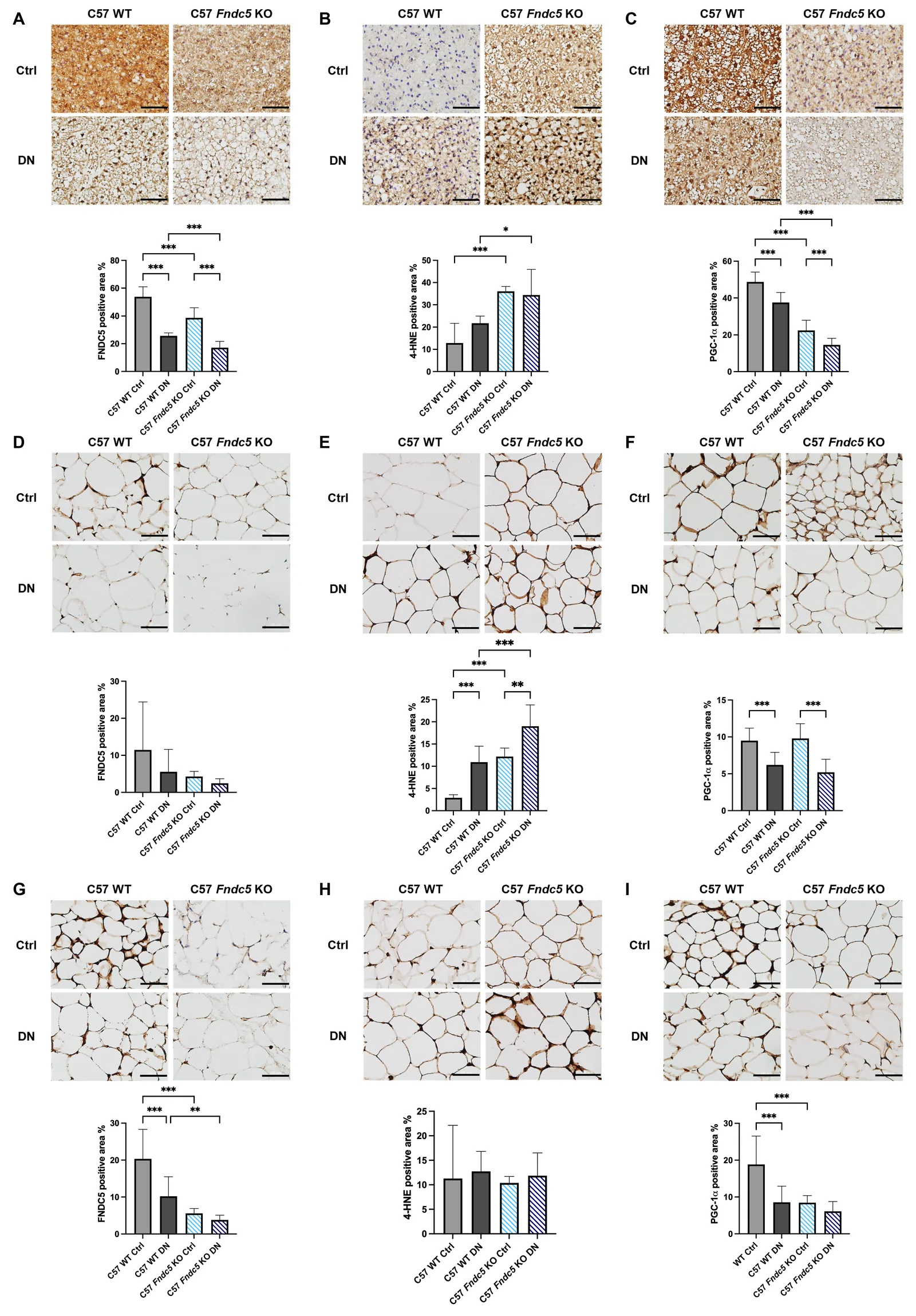
Morphological, IHC, and quantitative analyses of FNDC5 (A, D, G), 4-HNE (B, E, H), and PGC-1α (C, F, I) in different adipose tissues. Panels (A-C) show interscapular brown adipose tissue, (D-F) show epididymal white adipose tissue, and (G-I) show inguinal white adipose tissue. Each analysis compares WT and *Fndc5* KO groups, both with and without DN induction. Scale bars: 100 µm. Data are shown as mean ± SEM (n = 4 for each group). *P < .05; **P < .01; ***P < .001.

To investigate the systemic effects of *Fndc5* KO on metabolic tissues in DN mice, we evaluated lipid peroxidation and PGC-1α expression through IHC staining across multiple tissues, including the pancreas (**Fig. 4A-C**), muscle (**Fig. 4D-F**), and three types of adipose tissue: interscapular brown, epididymal white, and inguinal white adipose tissues (**Fig. 5**). Across all examined metabolic tissues, FNDC5 expression was notably reduced in C57 WT DN mice compared with C57 WT controls, with an even more pronounced decrease in *Fndc5* KO mice. In terms of lipid peroxidation and oxidative stress, we observed significantly elevated 4-HNE levels in the pancreas, muscle, and interscapular brown adipose tissue (**Fig. 5B**) in *Fndc5* KO DN mice compared with C57 WT DN groups, indicating heightened oxidative stress due to FNDC5 deficiency in these tissues. Additionally, epididymal white adipose tissue showed markedly elevated lipid peroxidation in C57 WT DN mice compared with C57 WT controls, with *Fndc5* KO DN mice exhibiting even higher 4-HNE levels, reflecting an augmentation of oxidative stress (**Fig. 5E**). However, no significant differences in 4-HNE expression were observed in the inguinal white adipose tissue across all groups (**Fig. 5H**). Regarding mitochondrial biogenesis, PGC-1α expression was significantly reduced in C57 WT DN mice compared with C57 WT controls in all metabolic tissues examined, with *Fndc5* KO DN mice showing an even more pronounced decrease in the pancreas, muscle, and interscapular brown adipose tissue (**Fig. 4C, Fig. 4F** and** Fig. 5C**). In contrast, PGC-1α levels in epididymal and inguinal white adipose tissues showed no significant differences between *Fndc5* KO DN and C57 WT DN groups (**Fig. 5F** and** Fig. 5I**), suggesting a variable impact of FNDC5 deficiency on mitochondrial biogenesis in different adipose tissues. These findings suggest that FNDC5 deficiency aggravates the oxidative stress and mitochondrial dysregulation in specific metabolic tissues under diabetic conditions.

## Discussion

In this study, we successfully established a DN model in C57 WT and *Fndc5* KO mice using a high-fat diet combined with STZ injection, as confirmed by elevated blood glucose levels and DN features, including proteinuria, glomerular basement membrane thickening, mesangial expansion and podocyte effacement. Interestingly, while *Fndc5* KO DN mice exhibited more severe histological damage, including podocyte effacement and mesangial expansion, their proteinuria remained comparable to the WT DN group at the 15-week mark. This 'structural-functional dissociation' suggests that at this specific time point, the kidney may still possess sufficient functional reserve to compensate for the accelerated morphological remodeling, suggesting that FNDC5 deficiency may primarily accelerate early structural injury, and a longer observation period might be required to witness the eventual divergence in overt clinical parameters such as ESKD. Furthermore, the relatively lower glycemic burden observed in *Fndc5* KO DN mice compared to WT DN mice may have further contributed to this delayed functional decline. It is noteworthy that the *Fndc5* KO mice had a lower induction rate of diabetes compared to the WT controls. This discrepancy may be attributed to the specific protocol of a single-dose STZ injection combined with high fat diet, or perhaps an inherent resistance in *Fndc5* KO mice against STZ-induced cytotoxicity.

Nevertheless, the *Fndc5* KO DN mice exhibited significantly more severe renal structural injury and mitochondrial disintegration despite having a lower cumulative glycemic burden, suggesting that FNDC5 is associated with a renoprotective effect. The impact of FNDC5 deficiency on lipid peroxidation was evident in kidney tissues, where significantly increased 4-HNE expression indicated elevated oxidative stress in FNDC5 deficient DN mice compared with WT DN mice. This was accompanied by a significant reduction in PGC-1α expression, suggesting impaired mitochondrial biogenesis and oxidative metabolism in the absence of FNDC5. Electron microscopy revealed mitochondrial abnormalities such as swollen mitochondria with inner membrane cristae disruption, especially pronounced in *Fndc5* KO DN mice. Furthermore, the elevated DNA damage in *Fndc5* KO diabetic kidneys, as shown by the TUNEL assay, highlights FNDC5's potential role in modulating cell survival under diabetic stress. Systemic analyses across metabolic tissues, including pancreas, muscle, and various adipose tissues, showed that FNDC5 deficiency is associated with aggravated oxidative stress and mitochondrial dysregulation, as indicated by significantly elevated 4-HNE and reduced PGC-1α levels. Together, these findings underscore the essential role of FNDC5 in preserving mitochondrial integrity and mitigating oxidative damage across systemic tissues under diabetic conditions, positioning FNDC5 as a potential therapeutic target to address metabolic and renal complications in diabetes.

Mitochondrial dysfunction emerges early in hyperglycemia and serves as a pivotal event in the progression of DN. In this study, the absence of FNDC5 was associated with aggravated diabetic kidney damage, as evidenced by significant mitochondrial injury and increased oxidative stress in *Fndc5* KO DN mice. This injury was further characterized by reduced PGC-1α expression, a key regulator of mitochondrial biogenesis and function, along with increased mRNA levels of *Dnm1l* and *Fis1*, markers of mitochondrial fission, and decreased mRNA levels of *Mfn1*, a marker of mitochondrial fusion. Additionally, the downregulation of ATP5B and ATP5A1 reflected a reduction in essential oxidative phosphorylation components, further reinforcing the molecular and structural mitochondrial impairment in this context 19. The imbalance in mitochondrial dynamics, characterized by increased fission and reduced fusion, is consistent with previous studies linking mitochondrial dysfunction to the pathogenesis of DN 8, 20. The dual role of mitochondrial fission in promoting the removal of damaged mitochondria via mitophagy and facilitating the proliferation of healthy mitochondria is crucial for cellular homeostasis. However, the increased expression of *Dnm1l* and *Fis1* in the absence of FNDC5 suggests enhanced mitochondrial fission, which may initially act as an adaptive response but, when dysregulated, contributes to persistent mitochondrial structural damage and dynamic imbalance 20. These findings highlight the critical role of FNDC5 in preserving mitochondrial integrity and mitigating oxidative stress, aligning with broader insights into mitochondrial dynamics in DN.

**Figure 6:**
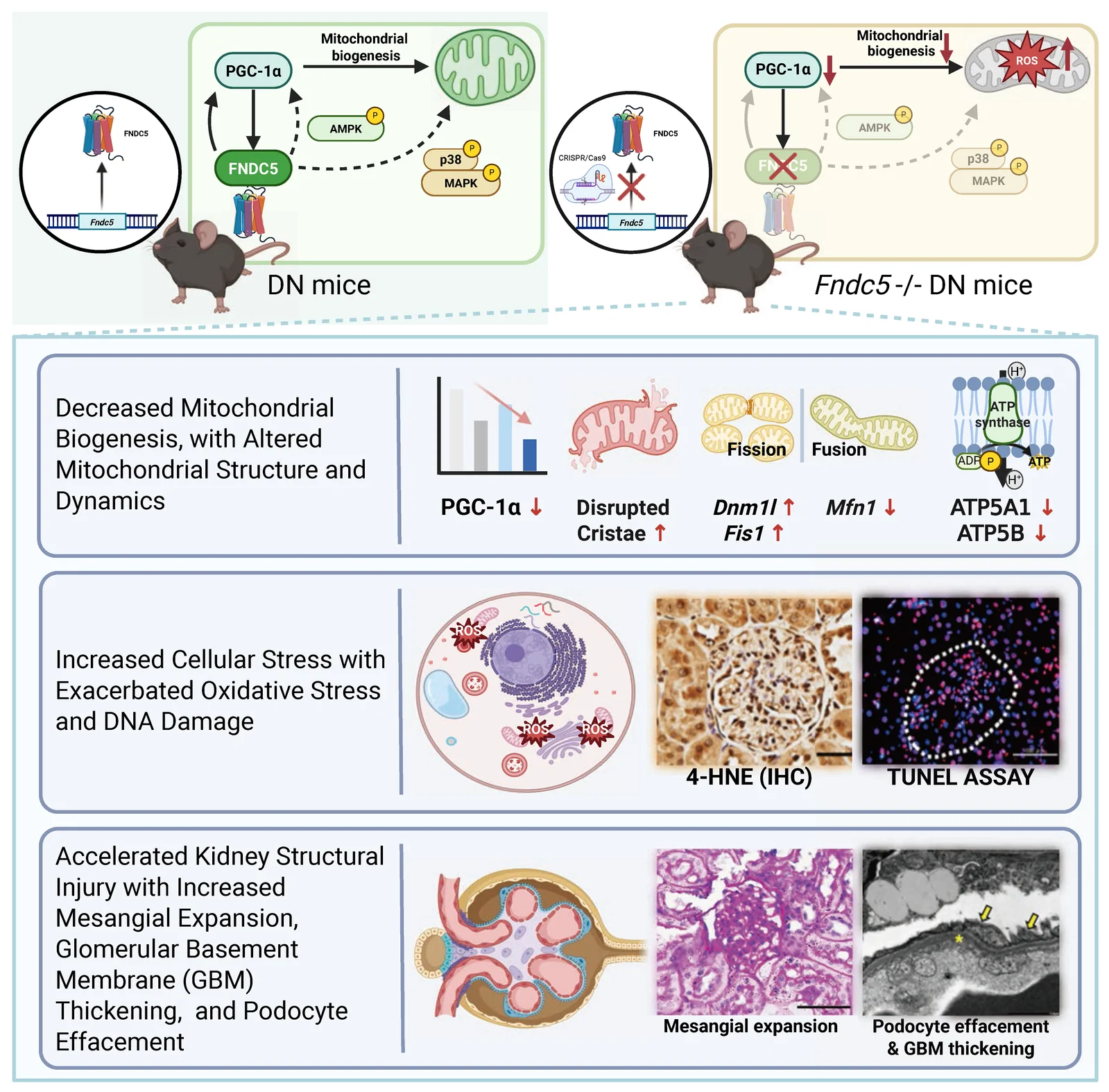
** Integrated schematic model linking FNDC5 to mitochondrial integrity in diabetic nephropathy (DN).** FNDC5 may sustain the expression of PGC-1α, which serves as its own upstream regulator, either directly or through an AMPK-mediated positive feedback loop. PGC-1α is a master regulator of mitochondrial biogenesis; additionally, FNDC5 may modulate mitochondrial function via the p38 MAPK axis. In FNDC5-deficient DN mice, the loss of FNDC5 and the subsequent decrease in PGC-1α levels impair mitochondrial homeostasis. This condition is characterized by structural alterations such as disrupted cristae, increased mitochondrial fission signaling (*Dnm1l* and *Fis1*), and reduced fusion signaling (*Mfn1*). Furthermore, expression of ATP synthase subunits ATP5A1 and ATP5B is reduced. These changes within the 'vicious cycle' of mitochondrial failure lead to fragmentation of mitochondrial networks, increased oxidative damage as shown by 4-HNE IHC, and DNA damage detected by TUNEL assay. Collectively, these cellular stressors accelerate the progression of DN, resulting in kidney structural injury characterized by mesangial expansion, glomerular basement membrane thickening, and podocyte effacement.

Our study found that increased lipid peroxidation and reduced PGC-1α expression were observed in metabolic tissues beyond the kidneys, including the pancreas, muscle, and adipose tissues, in *Fndc5* KO DN mice. We argue that FNDC5 is essential for the stabilization of its own upstream regulator, PGC-1α. This paradigm explains why the loss of FNDC5 results in a significantly more severe PGC-1α deficit than diabetes alone. While the precise upstream signaling pathways in renal cells remain to be fully elucidated, previous research in skeletal muscle has demonstrated that exercise stimulates AMPK phosphorylation, which in turn enhances PGC-1α expression 21. Interestingly, exogenous irisin treatment has also been shown to stimulate AMPK phosphorylation, leading to increased glucose uptake and suppressed glycogenolysis 22. Furthermore, irisin has been observed to directly upregulate Ppargc1a gene expression *in vitro* 23. Taken together, these findings suggest that FNDC5 may sustain PGC-1α expression either directly or through an AMPK-mediated feedback loop. The mitochondrial structural injury and dynamic disturbances observed in our model are closely linked to this PGC-1α deficit, given its master regulatory role in mitochondrial biogenesis. Moreover, considering the established link between FNDC5 and the p38 MAPK axis in other metabolic contexts, it is plausible that impaired p38 signaling also contributes to the observed mitochondrial disintegration 24. By framing our results within this 'vicious cycle' of mitochondrial failure, we provide a more robust logical foundation for the severe structural damage visualized by electron microscopy and the heightened DNA damage seen in the TUNEL assays (**Fig. 6**).

Current therapeutic strategies for DN primarily focus on lifestyle modification, renin-angiotensin system blockade therapy, and the management of glycemia and blood pressure 25. Targeting mitochondrial homeostasis offers a novel approach that could complement existing treatments 8. The use of glucose-lowering drugs such as glucagon-like peptide-1 receptor agonists and sodium-glucose cotransporter 2 inhibitors, which influence mitochondrial dynamics, further supports the potential of targeting mitochondrial pathways in kidney diseases 26. The role of mitochondrial oxidative metabolism in chronic kidney disease progression and the protective effects of antioxidants like avasopasem manganese in clinical trials also underscore the therapeutic potential of targeting mitochondrial pathways 27. Given the critical role of mitochondrial function in renal health, further studies are warranted to explore the underlying mechanisms of FNDC5 in mitochondrial regulation and its therapeutic potential in diabetes management. This could lead to the development of novel therapeutic strategies to enhance FNDC5 expression or function, thereby mitigating the complications of DN and improving overall metabolic health in diabetic patients.

Several limitations warrant consideration. First, the pooled analysis of male and female mice is a limitation of this study to elucidate potential sexual difference in FNDC5-mediated renoprotection. Second, although we confirmed the profound loss of FNDC5 expression across multiple metabolic tissues including the kidney, skeletal muscle, and adipose tissue, the lack of quantitative measurements for circulating irisin levels remains a limitation in clarifying the systemic bioactivity of this axis. Third, while our findings provide extensive evidence of mitochondrial structural disintegration and the dysregulation of fission/fusion dynamics, the extent of actual bioenergetic impairment, such as alterations in ATP production or respiratory capacity measured by Seahorse bioenergetic profiling, remains to be confirmed in future studies. Finally, the relatively small sample size in certain specialized assays may have limited the statistical power to reach significance in some parameters, despite the independent biological replicates providing consistent trends. Consequently, these observations should be interpreted with caution, and future studies with larger, sex-stratified cohorts are warranted to confirm these findings.

In conclusion, the study underscores the critical role of FNDC5 in maintaining mitochondrial integrity and protecting against oxidative stress and DNA damage in diabetic conditions, highlighting the potential of targeting FNDC5 pathways for developing novel therapeutic interventions for diabetes and DN complications.

### Author Contributions

**CWH, TMJ, YST** and** JSC** contributed to the study conception and design. **CWH** drafted the manuscript. **HHC**, **JMS**, **YST**, and **JSC** developed the methodology and contributed to the manuscript's writing, reviewing, and revising. **TMJ** and **CJL** acquired, analyzed, interpreted the data, and performed the statistical analysis. **YST** and **JSC** provided technical and material support. All authors read and approved the final manuscript. All authors read and approved the final manuscript.

## Supplementary Material

**Supplementary Method 1.** Generation of systemic C57BL/6JNarl-Fndc5^em1Jsc^ using CRISPR/Cas9 gene-editing technology. **Supplementary Method 2.** Composition of normal chow diet (Altromin 1310). **Supplementary Method 3.** Composition of High-Fat Diet (D12492). **Figure S1.** Diabetic induction and survival rates of C57 WT and C57 *Fndc5* KO mice. **Figure S2.** Impact of *Fndc5* KO on kidney and metabolic function in DN mice. **Figure S3.** The full uncropped and unedited versions of the Western blots for nephrin and podocin. **Figure S4.** The full uncropped and unedited versions of the Western blots for ATP synthase subunits (ATP5A1, ATP5B).

## Data Availability

Data generated and analyzed during this study are available from the corresponding author upon reasonable request.
