# Supplementary material for: CRISPR/Cas9-Driven Fndc5 Knockout Reveals Augmented Mitochondrial Structural and Dynamic Alterations in Diabetic Nephropathy
Source: Int J Med Sci. 2026 Jul 30;23(9):2884–98. doi: 10.7150/ijms.131007 (PMC13539912; doi:10.7150/ijms.131007)

Supplementary Material

Supplementary Methods and Figures

CRISPR/Cas9-Driven *Fndc5* Knockout Reveals Augmented Mitochondrial Structural and Dynamic Alterations in Diabetic Nephropathy

Chien-Wei Huang, Hsin-Hung Chen, Tzu-Ming Jao, Chia-Jung Li, Junne-Ming Sung, Yau-Sheng Tsai*, and Jin-Shuen Chen*

*These authors contributed equally to this work

Supplementary Method 1. Generation of systemic C57BL/6JNarl-Fndc5^{em1Jsc} using CRISPR/Cas9 gene-editing technology.

Supplementary Method 2. Composition of normal chow diet (Altromin 1310).

Supplementary Method 3. Composition of High-Fat Diet (D12492).

Figure S1. Diabetic induction and survival rates of C57 WT and C57 *Fndc5* KO mice.

Figure S2. Impact of *Fndc5* KO on kidney and metabolic function in DN mice.

Figure S3. The full uncropped and unedited versions of the Western blots for nephrin and podocin.

Figure S4. The full uncropped and unedited versions of the Western blots for ATP synthase subunits (ATP5A1, ATP5B).

Supplementary Method 1. Generation of systemic C57BL/6JNarl-Fndc5^{em1Jsc} using CRISPR/Cas9 gene-editing technology. (a) Schematic illustration depicting the genomic structure of the mouse *Fndc5* gene before and after CRISPR/Cas9-mediated deletion of exons 2 and 3. PCR primer set locations (5F, 3BWT, and 3BKO) used for genotyping WT and KO alleles are indicated by red arrows. Lightning bolts denote sgRNA/Cas9 target sites employed to generate systemic C57BL/6JNarl-Fndc5^{em1Jsc} KO mice. (b) Sequences and PCR conditions for primer sets (5F, 3BWT, and 3BKO) used for genotyping the WT and KO *Fndc5* alleles. Annealing temperature was set at 60°C. Expected PCR product sizes: WT allele (primers 5F+3BWT), 535 bp; KO allele (primers 5F+3BKO), 774 bp. (c) Representative PCR genotyping results for the *Fndc5* KO mice using two different primer combinations (5F+3BWT+3BKO and 5F+3BKO). Individual lanes represent mice with different genotypes: WT (+/+), heterozygous (+/-), and homozygous KO (-/-). WT alleles produce a PCR product of 535 bp (lower band), whereas KO alleles yield a larger PCR product of 774 bp (upper band). The primer pair 5F+3BKO specifically amplifies KO alleles only. DNA ladder (middle lane) is included for size reference. (d) Genomic DNA sequence of WT *Fndc5*, highlighting the sgRNA targeting sites (green boxes) and the corresponding Cas9 cleavage sites (indicated by red lines). Exon 2 and exon 3 sequences are labeled in red text. These sgRNA sequences were designed to specifically induce deletion of exons 2 and 3, generating the systemic *Fndc5* KO mouse model (C57BL/6JNarl-Fndc5^{em1Jsc}). KO, knockout; WT, wild-type.

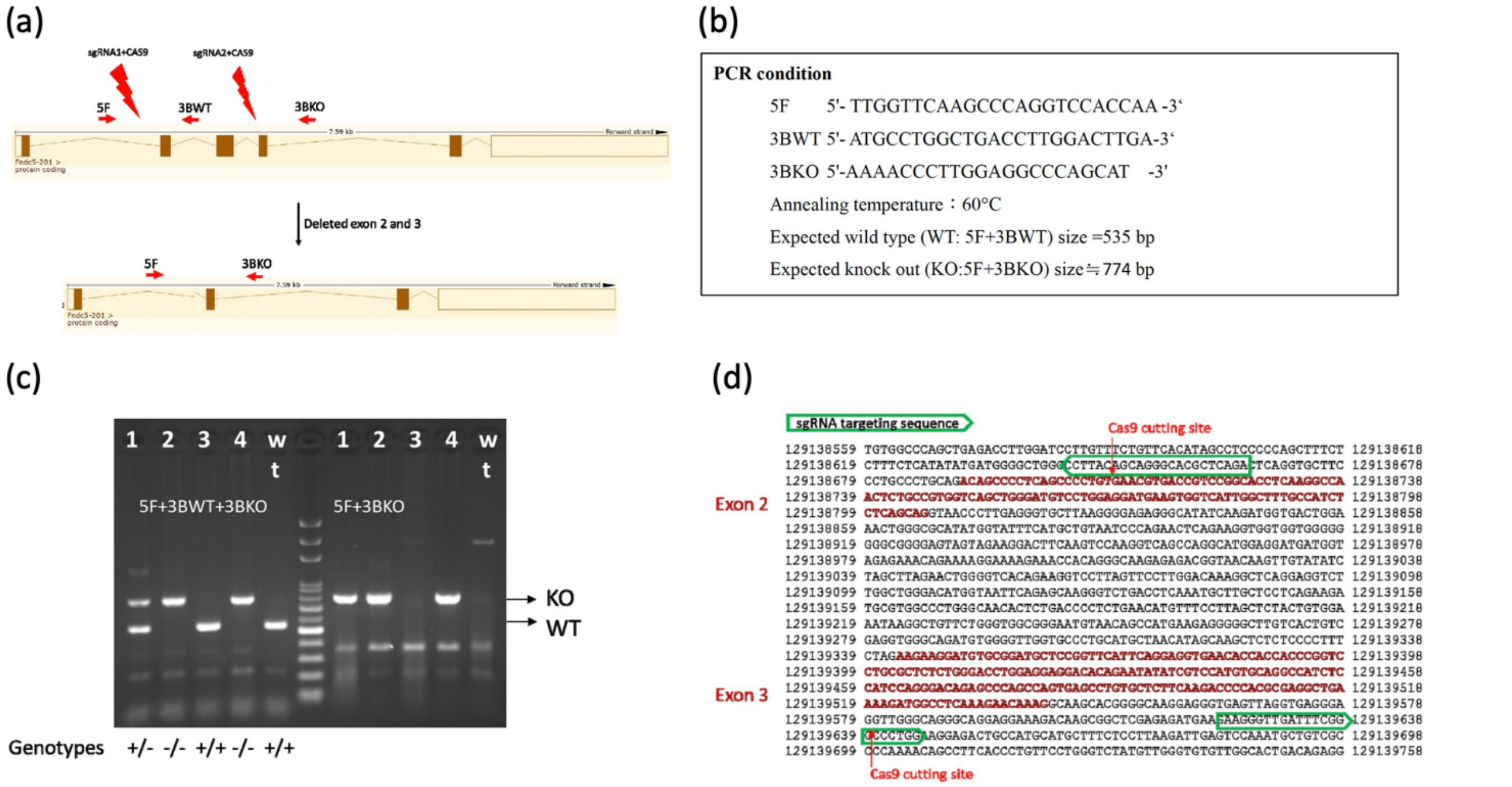

Supplementary Method 2. Composition of normal chow diet (Altromin 1310). The normal chow diet (Altromin 1310) contains defined proportions of protein (18%), carbohydrates (67.3%), and fats (4.3%), along with essential vitamins, amino acids, fatty acids, carbohydrates (mainly polysaccharides), minerals, and trace elements, formulated for rodents under standard laboratory.

Added vitamins

Content	Standard	Fortified	unit
Vitamin A	15,000	26,250	IU/kg
Vitamin D3	600	1,050	IU/kg
Vitamin E	77	133	mg/kg
Vitamin K3	3	5	mg/kg
Vitamin B1	18	32	mg/kg
Vitamin B2	12	21	mg/kg
Vitamin B6	9	16	mg/kg
Vitamin B12	24	42	µg/kg
Nicotinic acid	36	63	mg/kg
Pantothenic acid	21	37	mg/kg
Folic acid	2	4	mg/kg
Biotin	260	295	µg/kg
Choline chloride	600	1,050	mg/kg
Vitamin C	36	63	mg/kg

Amino acids

Content	Value	unit
Alanine	10,284	mg/kg
Arginine	14,822	mg/kg
Aspartic acid	21,735	mg/kg
Cystine	3,244	mg/kg
Glutaminc acid	43,649	mg/kg
Glycine	9,565	mg/kg
Histidine	5,508	mg/kg
Isoleucine	9,668	mg/kg
Leucine	17,123	mg/kg
Lysine	11,326	mg/kg
Methionine	3,171	mg/kg
Phenylalanine	10,549	mg/kg
Proline	13,955	mg/kg
Serine	11,300	mg/kg
Threonine	8,249	mg/kg
Tryptophan	2,929	mg/kg
Tyrosine	7,561	mg/kg
Valine	10,738	mg/kg

Fatty acid

Content	Value	unit
Arachidic acid C-20:0	148	mg/kg
Eicosanoic acid C-20:1	185	mg/kg
Alpha-Linolenic acid C-18:3	3,018	mg/kg
Linolenic acid C-18:2	21,996	mg/kg
Palmitic acid C-16:0	5,342	mg/kg
Stearic acid C-18:0	1,615	mg/kg
Oleic acid C-18:1	9,287	mg/kg

1310

Breeding diet for rats and mice

The 1310 formula is a cereal-based (soy, wheat, corn) fixed formula which is free of alfalfa and fish/animal meal and deficient in nitrosamines. This breeding diet was designed as complete feedingstuff for ad libitum feeding, recommended for breeding adult rats/mice and young rats/mice up to an age of 8 weeks.

Available as

1311 - Powder 1314 - 10mm pellets 1318 - Hybridpellet 1319 - Extrudate

Product variations

The 1310 formula is optionally also available in one or a combination of the following variants.

Variant P
deficient in phytoestrogens
Variant Fortified
autoclavable, increased vitamin content, coated on request
Variant Irradiated
irradiated with 5 to 50kGy
Variant TPF
total pathogen free
The contents may vary for these variants.

Metabolized energy

Content	Value		unit
Fat	463	(14%)	kcal/kg
Protein	901	(27%)	kcal/kg
Carbonhydrates	1,976	(59%)	kcal/kg

crude nutrients and moisture

Content	Value		unit
Moisture	111,241	(11.1%)	mg/kg
Crude Ash	61,128	(6.1%)	mg/kg
Crude Fibre	45,480	(4.5%)	mg/kg
Crude Fat	51,398	(5.1%)	mg/kg
Crude Protein	225,155	(22.5%)	mg/kg
Nitrogenfree extractives	505,599	(50.7%)	mg/kg

Carbonhydrates

Content	Value	unit
Monosaccharides	0	mg/kg
Disaccharides	54,151	mg/kg
Polysaccharides	350,300	mg/kg

Minerals

Content	Value	unit
Calcium	7,062	mg/kg
Potassium	10,144	mg/kg
Magnesium	2,055	mg/kg
Sodium	2,154	mg/kg
Phosphorus	5,090	mg/kg

Trace elements

Content	Value	unit
Aluminium	81.85	mg/kg
Chlorine	3,382.90	mg/kg
Iron	191.02	mg/kg
Flourine	3.05	mg/kg
Iodine	1.53	mg/kg
Cobalt	0.37	mg/kg
Copper	13.89	mg/kg
Manganese	77.69	mg/kg
Molybdenum	1.54	mg/kg
Sulfur	974.40	mg/kg
Selenium	0.26	mg/kg
Zinc	84.99	mg/kg

Supplementary Method 3. Composition of High-Fat Diet (D12492). The high-fat diet (D12492) used for the study consists of 60% kcal from fat, 20% from carbohydrates, and 20% from protein, providing a total of 5.24 kcal/g. Primary ingredients include casein (200 g/kg), L-cystine, maltodextrin, sucrose, and cellulose as dietary fiber. Fat content is predominantly provided by lard (245 g/kg) and supplemented by soybean oil (25 g/kg). Additionally, the diet includes essential minerals, vitamins, choline bitartrate, and specific colorants for product identification.

Product Data

DIO SERIES DIETS

OpenSource DIETS™

The "Original" High Fat Diets for Diet Induced Obesity

Formulas

Product #	D12450B		D12451		D12492	
	gm%	kcal%	gm%	kcal%	gm%	kcal%
Protein	19.2	20	24	20	26.2	20
Carbohydrate	67.3	70	41	35	26.3	20
Fat	4.3	10	24	45	34.9	60
Total		100		100		100
kcal/gm	3.85		4.73		5.24	
**Ingredient**	**gm**	**kcal**	**gm**	**kcal**	**gm**	**kcal**
Casein, 80 Mesh	200	800	200	800	200	800
L-Cystine	3	12	3	12	3	12
Corn Starch	315	1260	72.8	291	0	0
Maltodextrin 10	35	140	100	400	125	500
Sucrose	350	1400	172.8	691	68.8	275.2
Cellulose, BW200	50	0	50	0	50	0
Soybean Oil	25	225	25	225	25	225
Lard*	20	180	177.5	1598	245	2205
Mineral Mix S10026	10	0	10	0	10	0
DiCalcium Phosphate	13	0	13	0	13	0
Calcium Carbonate	5.5	0	5.5	0	5.5	0
Potassium Citrate, 1 H2O	16.5	0	16.5	0	16.5	0
Vitamin Mix V10001	10	40	10	40	10	40
Choline Bitartrate	2	0	2	0	2	0
FD&C Yellow Dye #5	0.05	0				
FD&C Red Dye #40			0.05	0		
FD&C Blue Dye #1					0.05	0
**Total**	**1055.05**	**4057**	**858.15**	**4057**	**773.85**	**4057**

Figure S1. Diabetic induction and survival rates of C57 WT and C57 *Fndc5* KO mice. (A) Diabetic induction rate in C57 WT and C57 *Fndc5* KO mice, shown by sex, with the number of mice with successful diabetic induction relative to the total number of mice in each group indicated above. (B) Survival rate at week 15 in diabetic C57 WT and C57 *Fndc5* KO mice, shown by sex, with the number of surviving mice relative to the total number of diabetic mice in each group indicated above.

A

Diabetes induction number / Total number

	C57 WT	C57 *Fndc5* KO
Male	5/6	8/9
Female	4/4	2/4

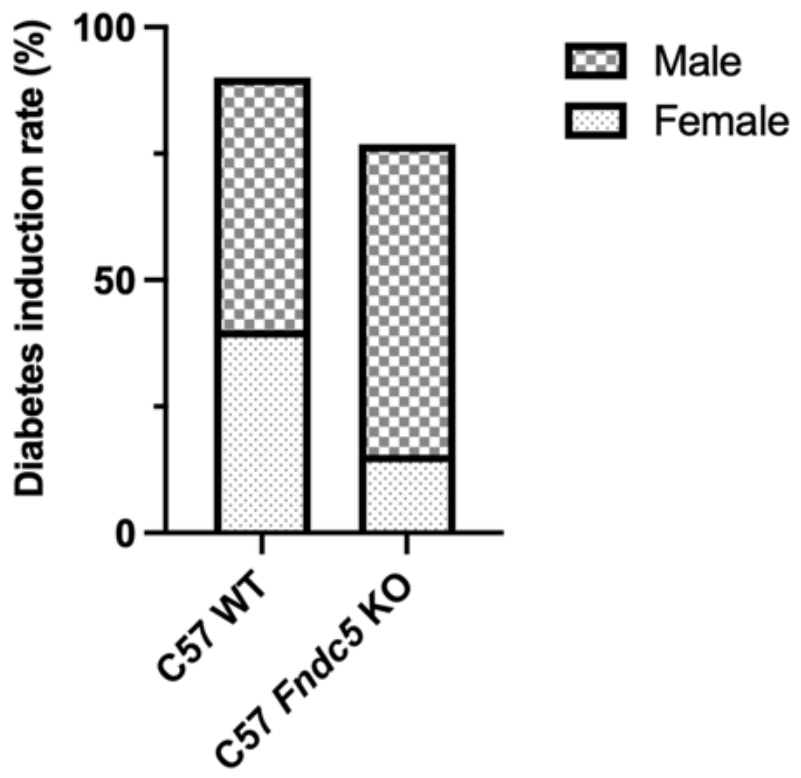

B

Surviving number / Diabetic mouse number

	C57 WT	C57 *Fndc5* KO
Male	3/5	5/8
Female	3/4	2/2

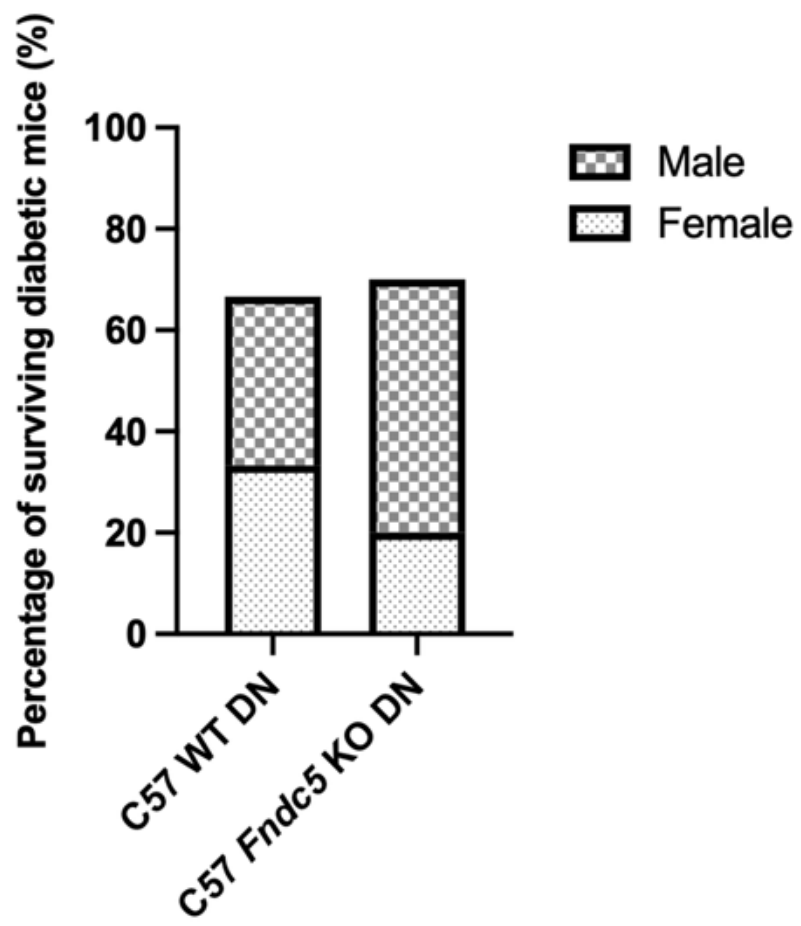

Figure S2. Impact of *Fndc5* KO on kidney and metabolic function in DN mice. (A) Kidney fibrosis was evaluated by Masson's Trichrome staining. Scale bars = 100 µm. Time course of blood urea nitrogen (B), serum creatinine (C), body weight (D), and serum triglycerides (E) in C57 WT and *Fndc5* KO mice, with and without DN induction. Data are shown as mean ± SEM.

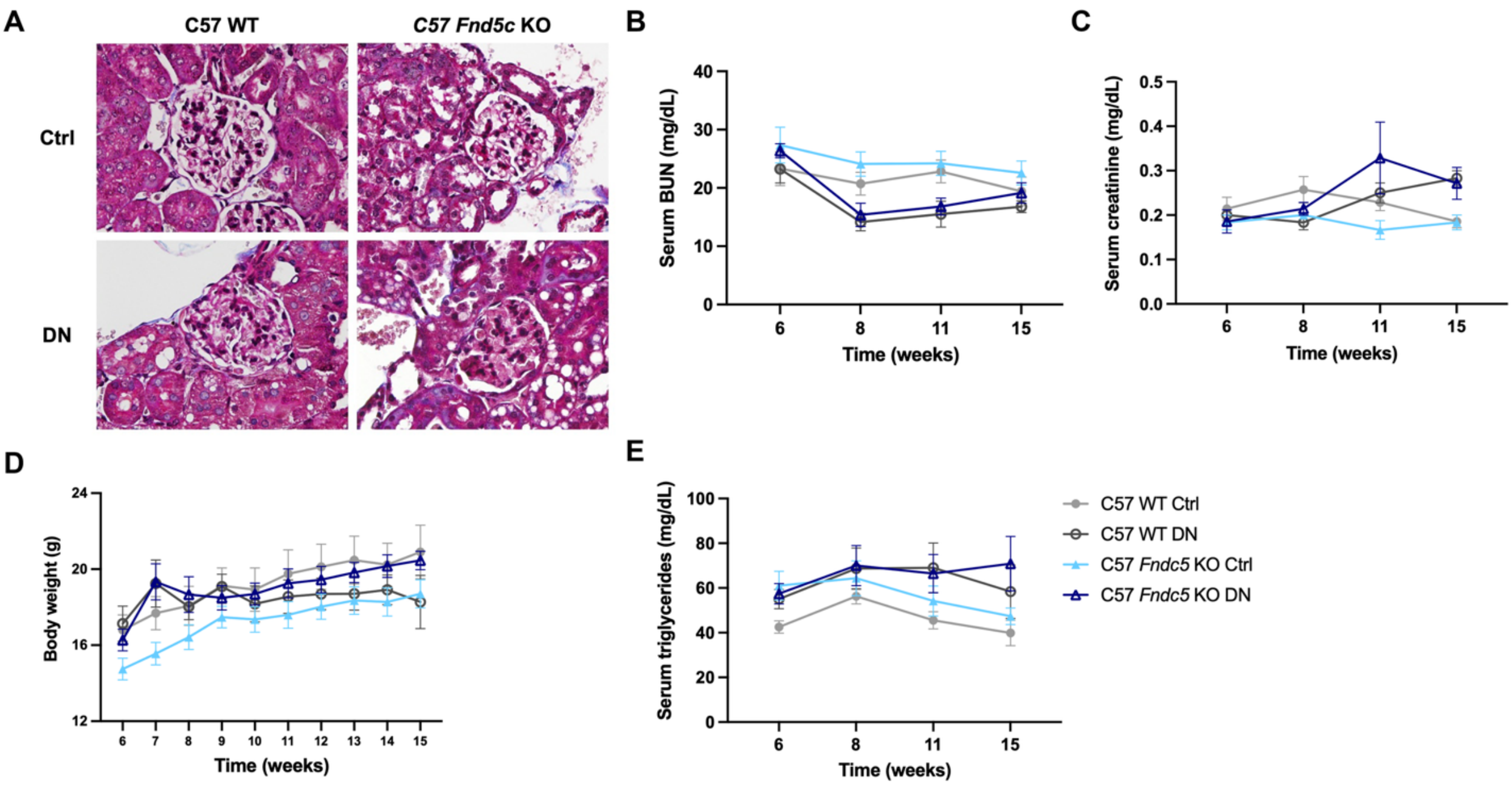

Figure S3. The full uncropped and unedited versions of the Western blots for nephrin and podocin. Original, unmodified Western blots showing nephrin, podocin and beta-actin as loading control. Red boxes indicate lanes used to construct Fig. 1F in the main manuscript.

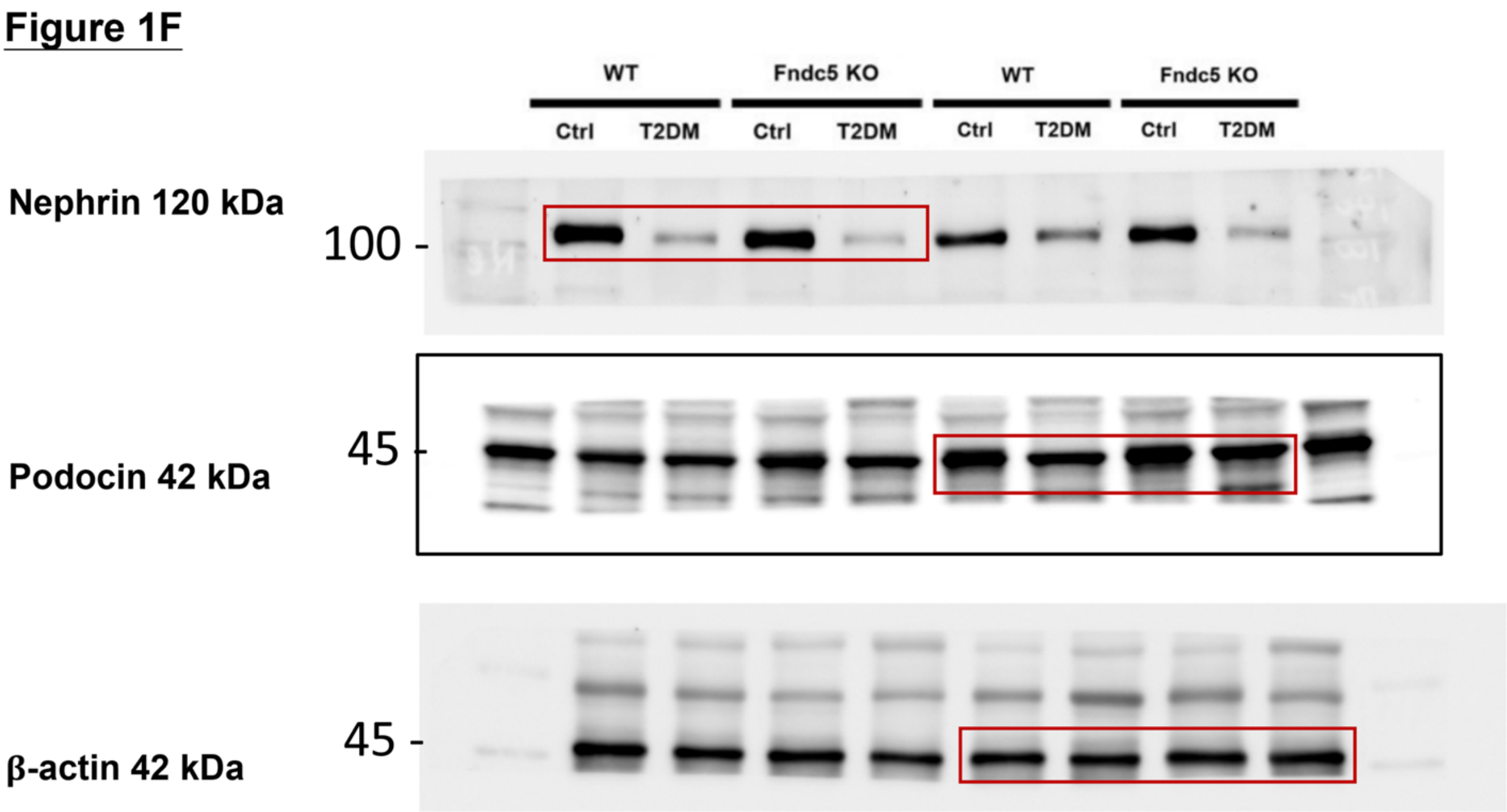

Figure S4. The full uncropped and unedited versions of the Western blots for ATP synthase subunits (ATP5A1, ATP5B). Original, unmodified Western blots showing ATP synthase subunits (ATP5A1, ATP5B) and beta-actin as loading control. Red boxes indicate lanes used to construct Fig. 3E in the main manuscript.

Figure 3E

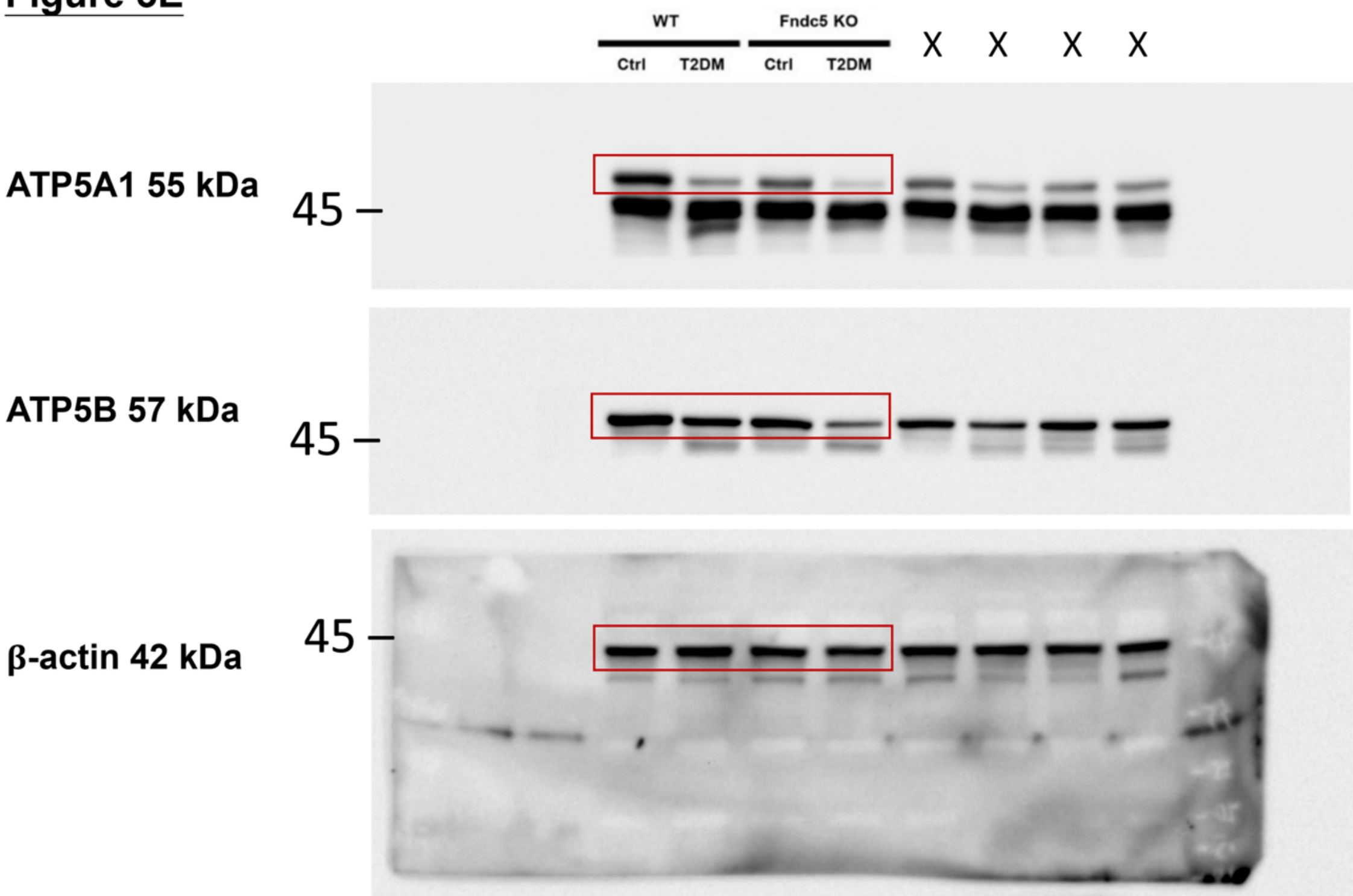

Supplement: Supplementary file 1 [file ijmsv23p2884s1.pdf]
